# A Practical Approach for High Precision Reconstruction of a Motorcycle Trajectory Using a Low-Cost Multi-Sensor System

**DOI:** 10.3390/s18072282

**Published:** 2018-07-14

**Authors:** Sarra Smaiah, Rabah Sadoun, Abdelhafid Elouardi, Bruno Larnaudie, Samir Bouaziz, Abderahmane Boubezoul, Bastien Vincke, Stéphane Espié

**Affiliations:** 1 SATIE Laboratory, University Paris Sud, 91405 Orsay, France; rabah.sadoun@g.enp.edu.dz (R.S.); abdelhafid.elouardi@u-psud.fr (A.E.); Bruno.larnaudie@u-psud.fr (B.L.); samir.bouaziz@u-psud.fr (S.B.); abderrahmane.boubezoul@ifsttar.fr (A.B.); bastien.vincke@u-psud.fr (B.V.); stephane.espie@ifsttar.fr (S.E.); 2 Signal and Communication Laboratory, National Polytechnic School, 16200 El-Harrach, Algiers, Algeria; 3 IFSTTAR, Champs-sur-Marne, F-77447 Marne la Vallée, France

**Keywords:** trajectory reconstruction, low-cost sensors, embedded systems, powered two wheels (PTW), safe trajectory, data fusion

## Abstract

Motorcycle drivers are considered among the most vulnerable road users, as attested by the number of crashes increasing every year. The significant part of the fatalities relates to “single vehicle” loss of control in bends. During this investigation, a system based on an instrumented multi-sensor platform and an algorithmic study was developed to accurately reconstruct motorcycle trajectories achieved when negotiating bends. This system is used by the French Gendarmerie in order to objectively evaluate and to examine the way riders take their bends in order to better train riders to adopt a safe trajectory and to improve road safety. Data required for the reconstruction are acquired using a motorcycle that has been fully instrumented (in VIROLO++ Project) with several redundant sensors (reference sensors and low-cost sensors) which measure the rider actions (roll, steering) and the motorcycle behavior (position, velocity, acceleration, odometry, heading, and attitude). The proposed solution allowed the reconstruction of motorcycle trajectories in bends with a high accuracy (equal to that of fixed point positioning). The developed algorithm will be used by the French Gendarmerie in order to objectively evaluate and examine the way riders negotiate bends. It will also be used for initial training and retraining in order to better train riders to learn and estimate a safe trajectory and to increase the safety, efficiency and comfort of motorcycle riders.

## 1. Introduction

Motorcycle drivers are considered the most vulnerable road users. In France, such riders account for more than 20% of all road fatalities (compared with 15% in Europe as a whole) and 43% of road injuries (ONISR, 2014). For the same distance traveled, the risk to be killed in a fatal crash is 35 times higher for a motorcyclist than for an automobilist [1]. More than 50% of individual motorcycle crashes are due to loss of control in bends (including crossroads and intersections) because of the complexity of motorcycle dynamics and the intrinsic instability of such vehicles. In 2012, in France, more than a third of all the powered two wheels (PTW) fatalities occurred in bends (248 killed).

Counter-measures are needed to improve road safety and decrease the risk of crashes in bends. In order to do so, it is necessary to better train riders to learn, estimate, and adopt a safe trajectory [2,3]. Motorcycle trajectory reconstruction represents a fairly important tool for an objective evaluation of bend taking practices and the characterization of the achieved trajectories compared to a safe trajectory.

The main idea of the safe trajectory is to “dive” towards the bend tangent point only when the driver sees the whole of the bend, up to its exit. This technique, if applied correctly, allows the rider to drive fast and safe because it allows him to anticipate dangers and potential hazards. Until today, no system exists to objectively evaluate bend-taking maneuvers. This evaluation is subject to an instructor’s “imprecise” appreciation of the French Gendarmerie.

In this context, the purpose of this work is to develop an algorithm using low-cost sensors to accurately reconstruct motorcycle trajectories in order to develop a tool for the objective evaluation of the achieved trajectory and that allow PTW riders to receive better training or retraining that focuses on “safe” cornering. The challenge is to achieve a high accuracy trajectory reconstruction by using a system that can be embedded implementing low-cost sensors and proposing an alternative solution to the traditional INS/GPS systems. The powered two wheels (PTW) used for this study is a motorcycle instrumented by the VIROLO++ Project team. Thanks to experienced and inexperienced (trainee) French national gendarmerie drivers, a very large dataset was acquired at the “La Ferté Gaucher” circuit by the VIROLO++ team [3,4].

The main contributions of our work are summarized below:

- A trajectory reconstruction study involving algorithms and ad hoc sensors. Bend trajectory reconstruction is highlighted namely for driver safety.
- Identification of an optimal low-cost system further to a multi-sensor instrumentation and different approaches for trajectory reconstruction, beyond simulation.
- An alternative solution to the INS/GPS systems, that allows a high-precision trajectory reconstruction. Results are given using a real dataset provided by various drivers.

This paper is organized in two major sections. The first one is dedicated to the literature survey, the description of the multi-sensor system used, the circuit-based experiments, and the major data adjustment. In the second section, we firstly explore and evaluate the usual methods of trajectory reconstruction. Then, we demonstrate the gain obtained with our proposed fusion method comparatively to the best of the usual method of reconstruction.

## 2. Literature Survey

Among the existing literature about motorcycle trajectory reconstruction, there is no work that addresses the problem of “safe trajectory”. In addition, motorcycle trajectory is usually reconstructed using traditional GPS/INS systems that can achieve good accuracy only if the GPS signal is available. However, during a GPS outage, the accuracy is degraded. Some works proposed an alternative solution based on vision systems, but the accuracy obtained is far from the expected objectives.

It is also worth noting that works about motorcycle trajectory reconstruction are limited compared to cars because the dynamics of a two-wheeled vehicle represents a higher level of complexity. In this section, we present research works that deal with motorcycle trajectory reconstruction.

In [5], Yuichiro Koyama presents a new algorithm (as he cited) for motorcycle trajectory reconstruction using GPS only. This algorithm is based on the interpolation of satellites positions that cannot provide adequate data within a few seconds (missing observation data) using polynomial curves calculated by the least-squares method and the calculation of the motorcycle coordinates based on interpolated pseudoranges. The obtained trajectory is smoothed using an extended Kalman filter. This method allowed obtaining a trajectory with an accuracy of less than one meter. This precision is obtained only if the observed data are absent for less than 7 s. No hardware realization was described.

Luca Gasbbaro et al. [6] presented an algorithm for the precise (as mentioned) reconstruction of the motorcycle trajectory based on vision integration and miniaturized MEMS accelerometers, using an extended Kalman filter and a dynamic model of the bike. The constraints of the model act as virtual measurements and make it possible to estimate the biases and drifts of the accelerometers. The visual reference marks were used to estimate the biases and drifts of the navigation sensors. The idea is to distribute a number, more than six, of accelerometers in specific locations on the chassis of the motorcycle and then use the shape invariance of the rigid body motion to estimate the trajectory. The data acquisition system uses a basic processing unit; a PC-104 industrial computer (266 MHz Geode, 256 MB RAM). A Compact Flash card was used to store the recorded data, as well as a Linux operating system and user’s programs.

The same idea involved in [6] was applied in [7], where the authors proposed a low-cost system based on micro electro-mechanical systems (MEMS) technology coupled with images through the Whipple model [8] and a cascade of a Kalman filter and a Bayesian particle filter to reconstruct the “Vespa” scooter trajectory. The authors used an Xsens MTi-G IMU, a 1.3 megapixel progressive scan color SONY CCD camera and a notebook computer for data acquisition. The reference trajectory was determined by a Novatel DL-4 double frequency GPS receiver. Their method provides relatively acceptable accuracy (mean error: 1.033 m, max error: 10.12 m, absolute mean: 3.2 m, and standard deviation: 2.53 m). However, the application of this method depends on the environmental conditions. The roll angle estimation in this work is based on the Hough transform, which necessitates a minimal amount of linear elements in the scene, and their absence can degrade the achievable results. For instance, a complex skyline and low contrast between the road segment and neighboring object can be problematic, even if not common.

In [9], the authors proposed an experimental low-cost differential GPS/MEMS-IMU system, using an extended Kalman filter approach in a loosely coupled mode to accurately (as cited) reconstruct the trajectory and the orientation of a motorcycle. The system performance was evaluated through a set of experiments using a motorbike-embedded MEMS-IMU (Xsens MTi), rigidly fixed to the GPS antenna. A low-cost mono-frequency GPS receiver (u-blox AEK4) was used with a dual-frequency GPS receiver from Javard as a reference. The proposed system provided an absolute position accuracy of 0.5 m and an orientation accuracy of 1°–2°.

The advantage of [5] is the accuracy of the trajectory reconstruction obtained using only a GPS. However, the developed algorithm is applied only to data lost within a few seconds (7 s according to authors). Beyond the given loss time, the efficiency of the interpolation is reduced and the accuracy is degraded.

In [6,7], authors succeeded in reconstructing the motorcycle trajectory using a system based on vision (camera), MEMS technology and a computer (industrial computer in [6] and a notebook PC in [7]) for data processing. This system is complex, not embeddable, and depends on the environmental conditions. Additionally, the obtained accuracy was not mentioned in [6], the maximum error achieved in [7] is 10.11 m.

In [9], a high accuracy was achieved (50 cm) using a GPS/INS integration algorithm. The traditional GPS/INS systems can achieve great accuracies when the GPS signal is available. However, during GPS outage the accuracy is degraded.

From an application point of view, the main objective of [5] is the simulation of the motorcycle dynamics to analyze the behavior of both the motorcycle and the rider in a virtual three-dimensional space that correlates strongly with real driving tests. In [6], the authors consider the trajectory reconstruction as estimation techniques for the full reconstruction of the dynamical vehicle state. Their algorithm is used in racing applications. In [7], the authors aim to identify the vehicle position in a mapping reference frame for driving directions and best-route analysis with significant accuracy. In addition, in [9] the designed system is used in sport applications. The precise trajectory reconstruction is used to determine tire slips of a motorcycle.

The challenge in our work is to study several motorcycle trajectory reconstruction algorithms in order to design an embedded system, with an optimal set of low-cost sensors that allows the reconstruction of the motorcycle trajectory with high precision without using GPS (due to drawbacks of signal loss). This system can then be used to better train riders to adopt a safe trajectory in order to reduce the risk of crashes in bends and to improve road safety.

## 3. Multi-Sensor System and Circuit-Based Experiments Description

A multi-sensor architecture and an embedded data-logger have been designed by the VIROLO++ Project team [4]. The system allowed acquiring and recording data related to the rider behavior, actions, and to the motorcycle dynamics [10]. The system architecture is based on a CAN (controller area network) bus that interfaces multiple sensors and a data logger. This architecture makes it possible to add or remove one or more devices (sensors or recorders), without interrupting tasks of the others nodes. This offers an essential flexibility in the development phase of the multi-sensor system.

### 3.1. Embedded Sensors

**Figure 1:**
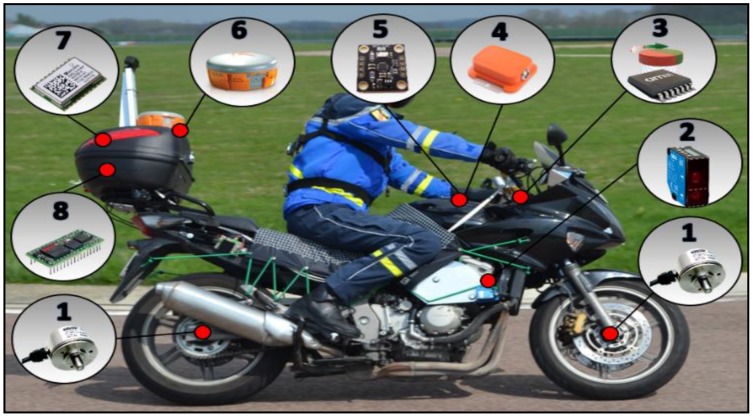
The ANR team instrumented motorcycle.

The instrumented PTW (Figure 1) integrates redundant sensors, “low-cost” sensors and “reference” sensors, in order to compare data measurements and to identify the subset of sensors necessary for each reconstruction method.

- Standard GPS receiver (7): a GPS module was designed, using an A2200-A circuit [11] of Maestro Wireless Solutions, to reduce the costs and to have a completely mastered GPS (sampling time: 200 ms);
- (10Dof) Inertial navigation system (5): The low-cost INS MPU9250 [12] of InvenSense was implemented in the motorcycle in order to acquire inertial movements and data required for the trajectory reconstruction (sampling time: 10 ms);
- Handlebar sensor (3): The magnetic sensor AS5047P of AMS is used to recover the absolute angle of the handlebar. The angle is coded on 14 bits which allows a maximum resolution of 2048 steps per complete rotation (0.176°/step) [13] (sampling time: 1 ms);
- Wheel tire sensors (1): Two Hall effect sensors in quadrature are attached to the front and rear wheels to measure the distance travelled by the motorcycle, used in trajectory reconstruction (sampling time: 1 ms);
- GPS RTK (6): The position delivered by the GPS RTK is used only as a reference to evaluate the accuracy of the reconstructed trajectory. The two (rover and base) GPS “Altus APS-3” of Septentrio are used to obtain real-time kinematic positioning [14] (sampling time: 40 ms);
- Inertial navigation system “Xsens” (4): The MTi Xsens [15] is used as a (redundant) reference system in order to check the degradation induced by others sensors. (sampling time: 10 ms); and
- Tilt sensors (2): To measure the roll angle, two identical (laser) optical distance sensors are placed on both sides (right and left) of the motorbike (sampling time: 10 ms).

### 3.2. Data Logger

**Figure 2:**
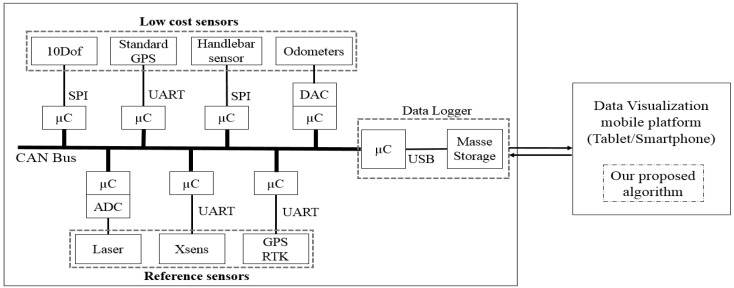
System architecture.

The data logger (recording node 8 in Figure 1) is based on a BECK programmable microcontroller and a CAN interface. The main function of the recorder is to collect messages sent on the CAN bus, put them in a specific format according to the identifier of the message, and place them in a specific file on the embedded memory storage. When receiving a CAN frame, the data logger writes a new block of data to a specific file containing the CAN frames and the reception times (time stamping task). The purpose of this instrumented architecture (Figure 2) is to have a variety of sensors in order to choose the optimal match algorithm sensors (allowing the best accuracy with low-cost sensors).

### 3.3. Circuit-Based Experiments

**Figure 3:**
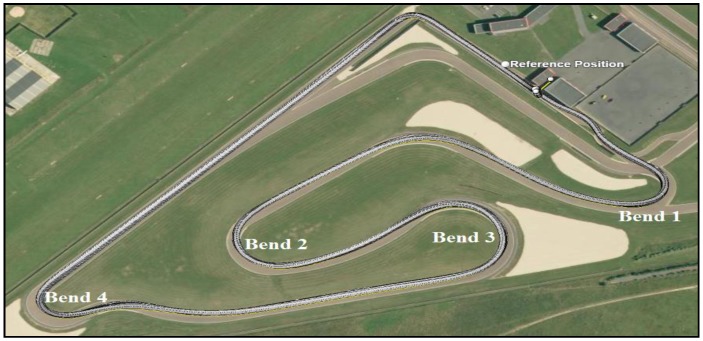
The “Ferté Gaucher” circuit mapped on IGN.

In order to evaluate the motorcycle trajectory reconstruction methods, several experiments were conducted. In this work, we have used the experiences carried out at the “La Ferté Gaucher” circuit (Figure 3) which is composed of different types of bends (right, left, 90°, and 180° bends with large or small curvature). The experimental data have been collected with a Honda CBF 1000 (Figure 1) which was driven on a 1.9 km loop. The departure point and the arrival point are identified. The motorcycle speed varies up to 110 km/h.

## 4. Sensor Data Correction

### 4.1. Reference Trajectory Correction

**Figure 4:**
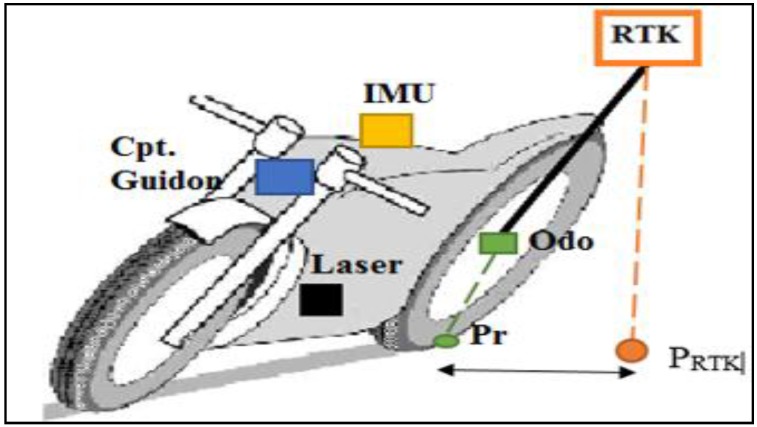
Sensor positioning.

Sensors were embedded in different positions on the motorcycle. As a consequence, the sensors’ measurements are in different references, especially in turning, as shown in Figure 4. Therefore, before evaluating the trajectory reconstruction methods, and in order to improve the accuracy, we considered the rear wheel contact point “*P_r_*” as a reference point and we brought all sensors’ data back to this reference point. Our reference frame, in this case, is the mobile triad (*P_r_*, *x*, *y*, *z*), specified by the Society of Automotive Engineers (SAE) [16]. The origin is established at the rear wheel contact point “$P_{r}$” with the road plane. The “*x*” axis is horizontal and parallel to the rear wheel plane. The “*z*” axis is vertical and directed downward while the “*y*” axis lies on the road plane. The road surface is, therefore, represented by the plane *z* = 0.

Positions of the sensors installed on the motorcycle are known. The GPS *RTK* position with respect to the reference point “$P_{r}$” is given by the coordinates (dx, dy, dz). Thus, the reference trajectory of the rear contact point “$P_{r}$” is calculated from the “*RTK*” trajectory using the following equation:

(1) $$\left[\begin{array}{c} X_{Pr}\\ Y_{Pr}\\ Z_{Pr} \end{array}\right]=\left[\begin{array}{c} X_{RTK}\\ Y_{RTK}\\ Z_{RTK} \end{array}\right]-R_{b}^{n}.\left[\begin{array}{c} dx\\ dy\\ dz \end{array}\right]$$

**Figure 5:**
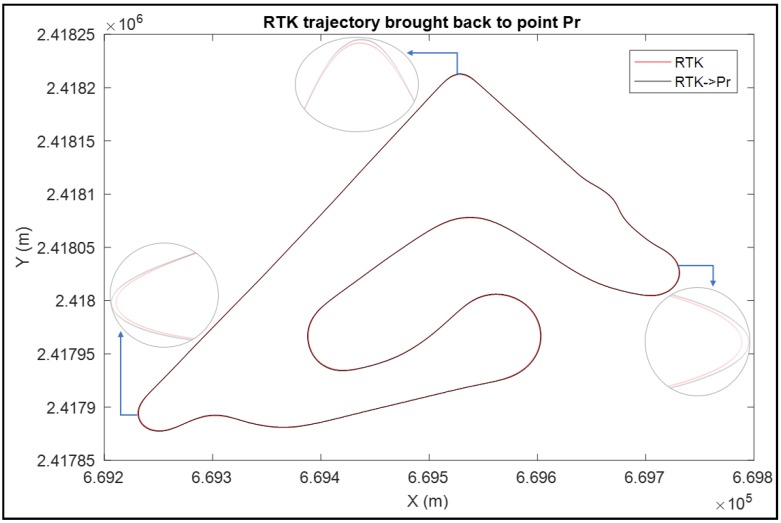
The reference trajectory brought back to the rear contact point.

Figure 5 shows the RTK reference trajectory before and after bringing it to the reference point.

It is clear that the main difference between the two trajectories exists in turns. When negotiating a bend, the motorcycle tilts to the inside bend direction. Thus, since the GPS RTK is installed on the bike trunk, its trajectory is always in the inside bend direction with respect to the rear contact point where the trajectory is in the outside bend direction.

### 4.2. Odometry Correction

Odometers are one of the sensors used to measure the motorcycle’s traveled distance independently from GPS. They work by counting wheel rotations and assume that the distance traveled is the number of wheel rotations times the tire circumference (tire diameter times pi).

According to our expertise, the wheel radius is not constant; it varies according to the roll angle. Therefore, a wheel radius model is required to correct the traveled distance estimated by odometers.

#### 4.2.1. Proposed Wheel Radius Model

**Figure 6:**
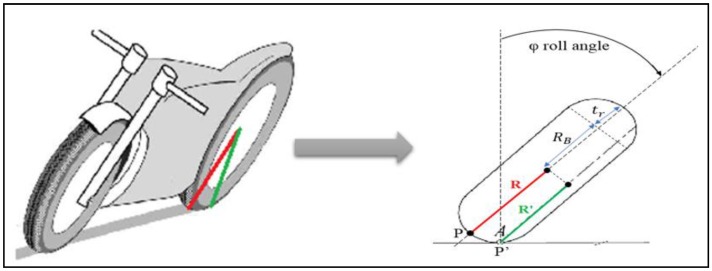
Lateral displacement of the rear contact point in a curve.

When negotiating a bend, the motorcycle passes from a vertical position to a tilted position with a roll angle “φ” in order to stay balanced. Following the roll motion, the contact point of the wheel with the road plane is displaced and the wheel radius is changed. Thus, in order to accurately estimate the motorcycle traveled distance, we propose the rear wheel radius model illustrated in Figure 6.

In a vertical position, the wheel radius of the motorcycle is *R*. However, when the motorcycle tilts, assuming a lateral roll without slippage on the road plane, the contact point of the rear tire “*P*” moves laterally, as illustrated in Figure 6, in the “*Y*” direction over a distance “$t_{r}\varphi$” which is proportional to the radius of the tire cross section “$t_{r}$” and the roll angle “φ” of the rear frame. The wheel radius in this case is *R*′:

(2) $$R^{\prime }=R_{B}-t_{r}\cos\left(\varphi \right)$$

where $R_{B}=\left(R-t_{r}\right)$: is the radius of the torus center circle.

#### 4.2.2. Validation of the Correction Model

**Figure 7:**
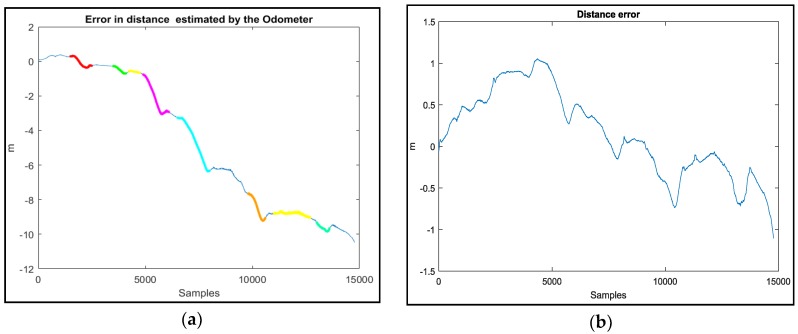
Difference between the real traveled distance and the one given by the odometers using a fixed wheel radius (**a**) and our proposed wheel radius model (**b**).

In order to validate our correction model, we measured the difference between the real traveled distance and the one given by the odometers using a constant wheel radius, Figure 7a, and using our proposed wheel radius model, Figure 7b. From Figure 7, it is clear that our proposed model greatly ameliorates the estimation of the distance and minimizes the error from (10 m) to (1 m with zero mean error).

**Figure 8:**
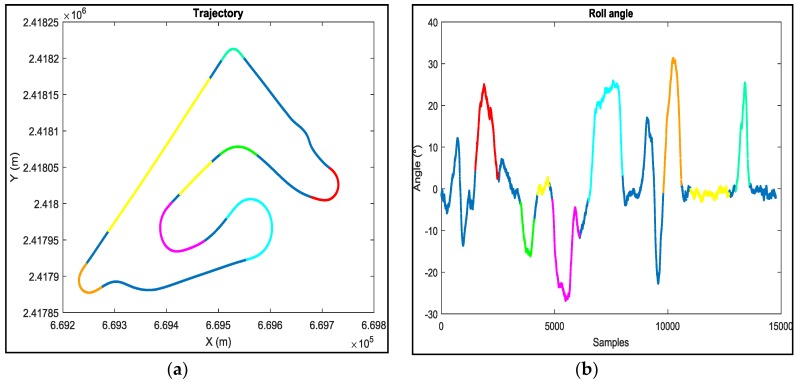
Location of the distance error (fixed radius) of each zone of Figure 7a on the trajectory (**a**) and the corresponding roll angle (**b**).

From Figure 8, we can clearly see that, in straight line trajectories, there is no difference between the real distance and the one estimated by the odometers using a fixed wheel radius, while in turns, where the roll angle is important, this difference increases (the colored zones, Figure 8) which confirms that the wheel radius changes according to the roll angle.

### 4.3. Data Preprocessing

**Figure 9:**
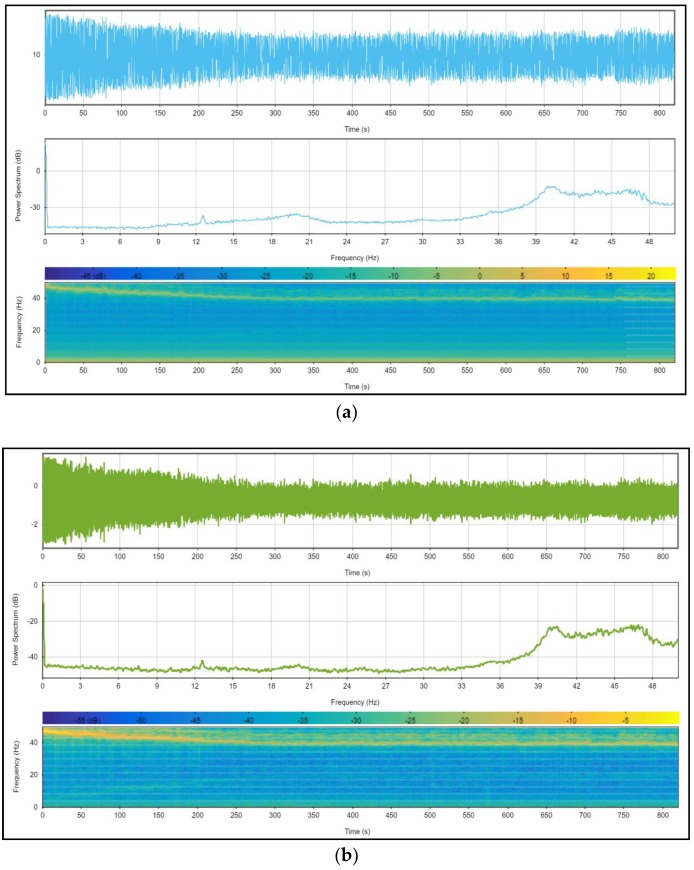
Illustration of spectrograms of “Ax” (**a**) and “Az” (**b**) IMU signals (signal on the top, power spectral density in the middle, and the signal spectrogram at the bottom).

During the data acquisition phase, no filter is applied [17] because the aim is to achieve a real dataset that can be used for other studies. Therefore, the sensors’ measurements are affected by noise (due mainly to engine vibrations) and a filtering step appeared necessary. In the first step, we proceeded to spectrally analyze the all IMU signals in static conditions (motorbike immobile and motor on). All the analyses exhibit two singular frequencies; less than 2 Hz and around 40 Hz. The two figures bellow (Figure 9a,b) illustrate our assertions.

Then, “wavelet” filter was chosen to denoise the data because of its advantage compared to conventional filters listed below.

- Little to no signal leakage or phase shifting of the original signal.
- The ability to denoise complex signals far better than conventional filters that are based on the Fourier transform.
- Wavelets are efficient for removing noise where the noise and signal spectra overlap. Conventional filters are efficient in removing out-of-band signals. However, if applied to in-band signals, wavelets will also remove the signal of interest.

To validate and confirm our choice, three filtering techniques were synthetized:

- Low-pass Butterworth filter with a 10 Hz cut-off (sixth order)
- Median filter (with a window of 20 points)
- Wavelet filter with a Daubechies mother wavelet of Db20. Six levels of decomposition have been considered as enough to provide a significant reduction of the high-frequency noise components.

Comparing the signal-to-noise ratio (SNR) of the three filtering techniques in Table 1, the “wavelet” has given better performances.

A static test was carried out in order to estimate and eliminate the static bias of sensors from the data before using them in the trajectory reconstruction algorithms.

## 5. Evaluation of the Usual Methods of PTW Trajectory Reconstruction: A Comparative Study

The developed multi-sensor architecture allows for evaluating several models of trajectory reconstruction. Depending on the nature of sensor information and the model used to reconstruct the motorcycle trajectories, we can distinguish four methods: kinematic models, absolute localization, relative localization, and data fusion-based localization.

### 5.1. Kinematic Model

**Figure 10:**
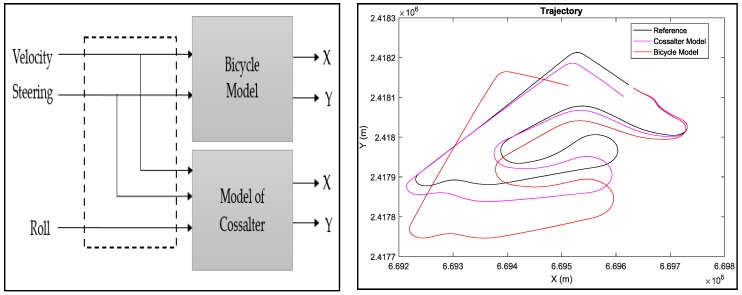
Trajectory reconstruction from kinematic models.

Several motorcycle kinematic models exist in the literature and give the position of the motorcycle according to a certain number of input data (sensor data). Among these models, we note the Cossalter model [16] and the bicycle model [17] (Figure 10).

In the literature, no dynamic model allowing the passage of the motorcycle position or the trajectory reconstruction exist, a reason why we used only the kinematic models.

### 5.2. Data Fusion Methods

This approach consists of a fusion of sensor data that presents measurement uncertainties for a “sufficiently accurate” positioning. Different approaches exist in the literature to fuse data delivered by sensors. A very good bibliographical study is presented in [18].

This solution is based on the idea of jointly using both localization methods: relative and absolute poses in order to take advantage of the complementarity of proprioceptive and exteroceptive sensors. Indeed, the absolute localization system is generally dedicated to regularly correct the estimate of the relative localization system in order to remedy the drift problems encountered with it. Dead reckoning provides measurements at a very high frequency, but requires initialization. Absolute localization provides long-term precision, but it suffers from problems of availability, latency and, often, insufficient frequency for some applications. Therefore, to reap the benefits and complementarities of the two localization systems, researchers paired these two families, giving birth to the data fusion approach like GPS/INS systems.

#### 5.2.1. GPS/INS Data Fusion

**Figure 11:**
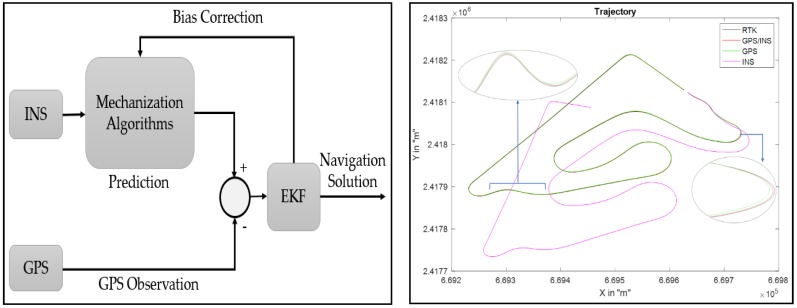
Loose coupling integration scheme (INS/GPS).

In the literature, several techniques are suggested to fuse GPS and INS data. The Kalman filter is the most used algorithm with, typically, three main strategies: namely loose integration, tight integration, and deep (or ultra-tight) integration. In this work, we have chosen to use a loosely-coupled implementation mode in a closed loop (Figure 11). This mode allows control of the navigation accuracy and reduces the cost of the design [19].

Due to the non-linearity of the process model, an extended Kalman filter of 15 states was built in this work using the dynamic equations of the error (the filter update is based on an error state vector which includes error vectors for position, velocity, attitude, accelerometer bias, and gyrometer bias, as explained in [20]).

In this method, the GPS measurements are used to correct data of the INS and to eliminate bias and drifts. However, the GPS accuracy is degraded because of multiple routes and for the small number of visible satellites (low availability). Sometimes GPS data are absent for a long time (if the satellites visibility conditions are degraded, the reception of the signals is blocked), which affects the accuracy of this method.

#### 5.2.2. INS/Odometer Data Fusion

**Figure 12:**
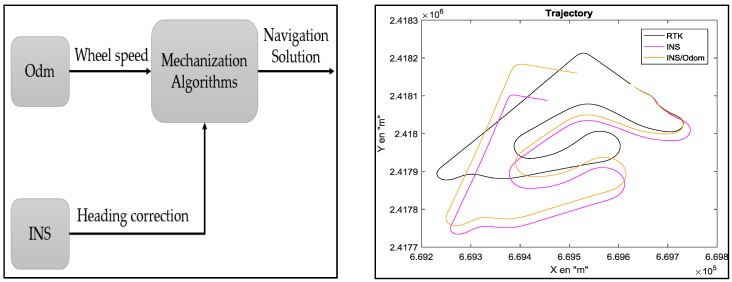
Schematic diagram of the INS/Odo integration.

In the literature [21,22], different configurations are proposed to integrate odometer and INS data. In our work, we have chosen the RISS configuration (reduced inertial sensor system) (Figure 12). 

The discrete form of the mechanization algorithm of this system is:

(3) $$\begin{array}{c} x\left(k+1\right)=x\left(k\right)+T_{e}\;V\left(k\right)\;\cos\left(\theta \left(k\right)\right)\\ y\left(k+1\right)=y\left(k\right)+T_{e}\;V\left(k\right)\;\sin\left(\theta \left(k\right)\right)\\ \theta \left(k+1\right)=\theta \left(k\right)+T_{e}\;W_{z} \end{array}$$

where $W_{z}$ is the gyroscope measurement (rad/s).

Two main errors influence the trajectory reconstruction:

- odometer errors, which come from the inaccuracy of the vehicle’s geometrical parameters; and
- heading errors, which come from the drifts in the gyroscopes data (accumulation of errors during the integration of the gyrometer’s data).

From Table 2, it is clear that the GPS/INS data fusion solution gives a precise reconstruction and good accuracy. However, this accuracy is obtained in good conditions (no GPS outage and the number of visible satellites is more than four). Contrariwise, during GPS outage, the accuracy of the reconstructed trajectory will strongly decrease. Therefore, in order to propose an alternative to the GPS/INS solution, we propose in this work to improve the INS/odometer data fusion method rather than kinematic models for two reasons:

- The first objective is the design of a motorcycle trajectory reconstruction system with an optimal set of low-cost sensors. In other words, we want to develop an algorithm that uses as few sensors as possible: in kinematic models three sensors (steering, roll, and odometer) are used, while in the INS/odometer method, only two sensors are involved (INS and odometer).
- The second objective is the use of a non-invasive approach. In our work, we aim to propose an algorithm that can be used and implemented without changing the basic design of the motorcycle. Thus, the INS/odometer algorithm can be used either directly if the commercial PTW contains an M-ABS or MTC, or by adding low-cost sensors. While in the case of kinematic models, adding a steering sensor may require some changes to the handlebar.

## 6. Our Proposal: Enhanced INS/Odometer Data Fusion

The trajectory reconstruction in the INS/GPS method is mainly based on two parameters, as illustrated in Equation (12): heading angle (yaw) and the traveled distance. In this work:

- in order to accurately estimate the traveled distance, the odometer measurements are improved using the wheel radius model that we proposed in Section 4.2; and
- in order to accurately estimate the heading angle, we propose to use the “INS” with the “Madgwick filter” algorithm [23].

The Madgwick filter is based on a quaternion representation, allowing the use of accelerometer and magnetometer measurements in an analytically-derived and optimized gradient descent algorithm to compute the direction of the gyroscope measurement errors as a quaternion derivative and to accurately estimate the attitude of the moving object.

In ideal conditions, i.e., absence of noise and magnetic deviation, the relation between the acceleration in the Earth frame “$a^{E}$” and the acceleration in the sensor frame “$a^{S}$” is given by Equation (4):

(4) $$a_{q}^{S}=q^{-1}⊗a_{q}^{E}⊗q$$

where:

- ⊗: is the quaternion multiplication.
- $a_{q}^{S}$: is the quaternion form of “$a^{S}$”, which can be written such as: $a_{q}^{S}={\left[\begin{array}{ccc} 0 & a_{x}^{S} & a_{y}^{S} \end{array}a_{z}^{S}\right]}^{T}$
- $a_{q}^{E}$: is the quaternion form of “$a^{E}$”. In static cases, $a_{q}^{E}={\left[\begin{array}{ccc} 0 & 0 & g \end{array}\right]}^{T}$ where g is the acceleration due to the gravity at the Earth’s surface ($g\approx 9.8\;m.s^{-2}$).

The relation between “$m^{E}$” and “$m^{S}$” is as follows:

(5) $$m_{q}^{S}=q^{-1}⊗m_{q}^{E}⊗q$$

where:

- $m_{q}^{S}$: is the quaternion form of “$m^{S}$”, which can be written such as: $m_{q}^{S}={\left[\begin{array}{ccc} 0 & m_{x}^{S} & m_{y}^{S} \end{array}\;m_{z}^{S}\right]}^{T}$
- $m_{q}^{E}$: is the quaternion form of “$m^{E}$”.

The kinematic equation of a rigid body that describes the variation of the attitude in terms of the quaternion, defined from the angular rate measurements delivered by the gyroscope, is given by the following equation:

(6) $$\dot{q}=\frac{1}{2}q⊗\omega_{q}^{S}$$

where “$\omega_{q}^{S}$” is the quaternion form of “$\omega^{S}$”.

**Figure 13:**
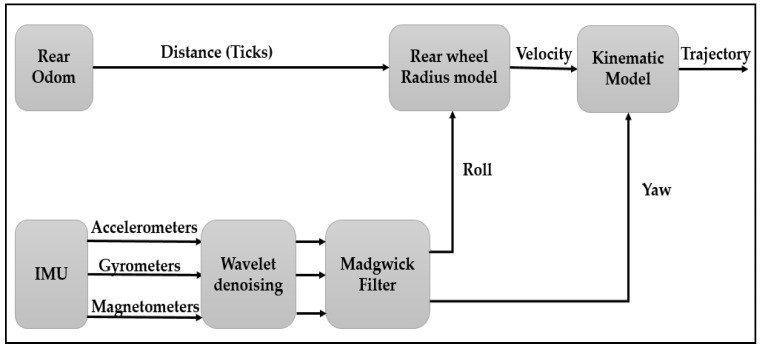
Schematic diagram of the new INS/odometer integration model.

We have chosen the Madgwick filter (Algorithm 1) rather than Kalman algorithm because their performances were compared in [23] and the results indicate that the Madgwick filter reaches levels of accuracy exceeding that of the Kalman algorithm; <0.6° static RMS error, <1.7° dynamic RMS error. Hence, the new system model of the INS/odometer integration is depicted in Figure 13.
**Algorithm 1.** Gradient descent-based orientation filter.$${\hat{h}}_{q,t}^{E}={\hat{q}}_{t-1}⊗m_{q,t}^{S}⊗{\hat{q}}_{t-1}^{-1}$$$${\hat{m}}_{q,t}^{E}={\left[\begin{array}{ccc} 0 & 0 & \sqrt{{\left({\hat{h}}_{x,t}^{E}\right)}^{2}+{\left({\hat{h}}_{y,t}^{E}\right)}^{2}} \end{array}\;{\hat{h}}_{z,t}^{E}\right]}^{T}$$$$F_{t}=\left[\begin{array}{c} {\hat{q}}_{t-1}^{-1}⊗a_{q,t}^{E}⊗{\hat{q}}_{t-1}-a_{q,t}^{S}\\ {\hat{q}}_{t-1}^{-1}⊗m_{q,t}^{E}⊗{\hat{q}}_{t-1}-m_{q,t}^{S} \end{array}\right]$$${\hat{q}}_{e,t}=J_{t}^{T}F_{t}$, where $J_{t}$ is the Jacobian matrix of $F_{t}$.
$${\hat{\omega }}_{e,t}^{s}=2{\hat{q}}_{t-1}⊗{\hat{q}}_{e,t}$$
$${\dot{\hat{\omega }}}_{b,t}^{S}=\omega_{e,t}^{S}$$
${\hat{\omega }}_{t}^{S}=\omega_{t}^{S}-\zeta^{S}\omega_{b,t}$ where $\zeta^{S}$ is the integral gain
$${\dot{\hat{q}}}_{t}=\frac{1}{2}{\hat{q}}_{t-1}⊗{\hat{\omega }}_{q,t}^{S}-\beta \frac{{\hat{q}}_{e,t}}{∥{\hat{q}}_{e,t}∥}$$
β is the divergence rate of $q_{t}$ expressed as the magnitude of quaternion derivative corresponding to the gyroscope measurement error. $q_{e},\omega_{e}$ are the quaternion and angular rate errors.


**Figure 14:**
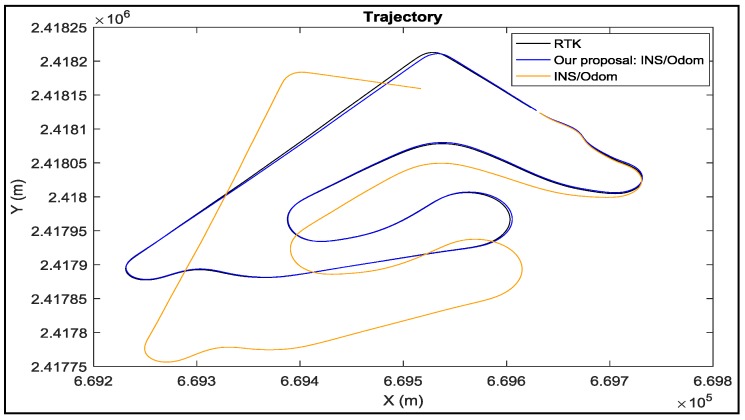
The results of our proposal compared to the traditional method INS/odo.

Figure 14 represents the results of the INS/odometer data fusion method before and after the improvements that we propose. From this figure, we can clearly see that the approach we proposed greatly ameliorates the trajectory reconstruction, especially in bends.

From Table 2 and Figure 14, it is clear that the integration of GPS/INS data and the proposed integration of odometer/INS data methods give the best reconstruction.

We are mainly interested in the reconstruction of bends instead of the whole trajectory in order to evaluate the behavior of the driver in bends and to compare it with a “safe trajectory”. For this reason, the second step consisted of evaluating the accuracy of the motorcycle trajectory in bends achieved by the proposed method and the traditional “GPS/INS integration” method. The two algorithms were tested to reconstruct bends, as shown in Figure 3, with six drivers. Each driver realized three complete trajectories (in total, 18 are achieved). Three indices were used for the quantitative evaluation [5]: bias error, error variance, and maximum error.

The three indices are defined by the following equations:

(7) $$\mathrm{Bias}\;\mathrm{error}=\sqrt{E_{x}^{2}+E_{y}^{2}}$$

(8) $$E_{x}=\frac{1}{t_{\max}}\sum_{t=1}^{t_{\max}}\Delta x\left(t\right)\;\mathrm{and}\;E_{y}=\frac{1}{t_{\max}}\;\sum_{t=1}^{t_{\max}}\Delta y\left(t\right)$$

where Δx(t) and Δy(t) are the positioning error in the east and north directions at epoch *t* ($t=1,\;2,\;\ldots ,t_{\max})$ respectively and:

(9) $$\mathrm{Error}\;\mathrm{variance}=\sqrt{V_{x}+V_{y}}$$

(10) $$V_{x}=\frac{1}{t_{\max}}\sum_{t=1}^{t_{\max}}{\left(\Delta x\left(t\right)-E_{x}\right)}^{2}\;\mathrm{and}\;V_{y}=\frac{1}{t_{\max}}\sum_{t=1}^{t_{\max}}{\left(\Delta y\left(t\right)-E_{y}\right)}^{2}$$

(11) $$\mathrm{Maximum}\;\mathrm{error}=\max\left[\sqrt{\Delta x{\left(t\right)}^{2}+\Delta y{\left(t\right)}^{2}}\right]$$

**Figure 15:**
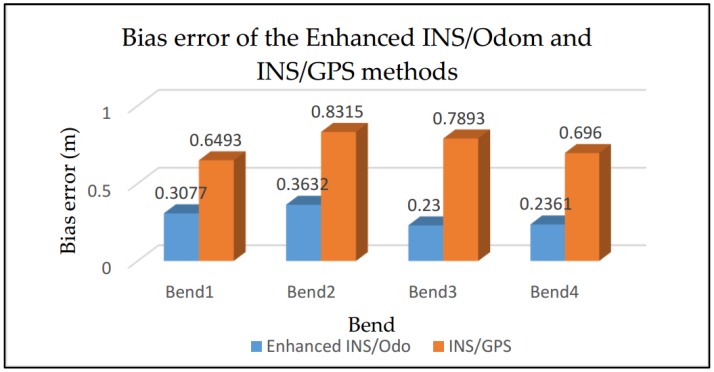
Bias error comparison.

According to Table 3 and Figure 15, the proposed method is more efficient than the traditional INS/GPS system. The accuracy of the proposed system is equal to the accuracy of a DGPS. The accuracy of the reconstruction is evaluated according to variance of the error, i.e., the precision varies between “Bias error ± Error variance”. The accuracy obtained with the proposed method varies between 23 cm ±25.20 cm and 36.32 cm ±31.38 cm with a maximum error of 89.41 cm obtained in bend 2 while the accuracy obtained using the INS/GPS integration varies between 64.93 cm ±34.56 cm and 83.15 cm±44.62 cm with a maximum error of 1.3085 m obtained in bend 4. An improvement of 61% in the accuracy is achieved compared to the INS/GPS method.

**Figure 16:**
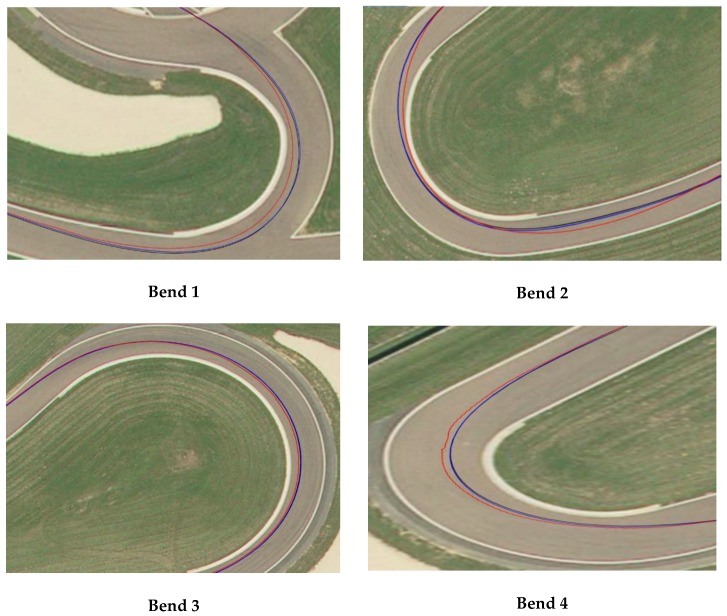
Comparison between the reference trajectory (black curve), GPS/ INS solution (red curve), and the proposed method (blue curve) in the four bends (Bend 1 to 4) showed in Figure 3.

Figure 16 shows bend measurement using the GPS/INS method (red curve) and its measurement using the proposed INS/odometer system (blue curve) in “Géoportail” compared to the reference bend given by “GPS RTK” (black curve). We can distinguish that the proposed method gives higher accuracy than the standard GPS/INS method.

## 7. Conclusions and Perspectives

In this paper, a comparative study of motorcycle trajectory reconstruction involving algorithms and ad hoc sensors was realized at a sampling time of 10 ms. Several methods and algorithms were evaluated in order to identify an optimal low-cost system further to a multi-sensor instrumentation for an accurate motorcycle bend reconstruction with high accuracy. We provide an experimental setup with a precise ground truth obtained through a GPS RTK.

An enhanced navigation system was proposed using INS/odometer data fusion combined with a Madgwick filter and a wheel radius calculation. The proposed approach represents a good alternative to the traditional INS/GPS system, especially during GPS outage where the accuracy of the GPS/INS solution is degraded. The results are given using a real dataset provided by different drivers.

The obtained accuracy is equal to that of a DGPS. However, the DGPS suffers from several problems:

- Poor dynamic characteristics: the GPS has a low frequency, thus, it provides the state information at low update rates.
- Low availability: accuracy is degraded for a small number of visible satellites (error can achieve 10 m).
- Data latency.
- Multipath errors: these errors occur when the GPS signal is reflected by objects such as large buildings or large areas of obstacles before it reaches the receiver antenna which increases the signal propagation time. This causes an overvaluation of the flight time and, therefore, generates positioning errors.

Our approach overcomes all of these problems and ensures accurate results at high frequency, availability, and solution continuity, which allows to objectively evaluate bend-taking maneuvers and to better train riders to adopt a secure bend.

The designed system will be used by the French Gendarmerie in order to objectively evaluate bend-taking practices. High precision is required to accurately reconstruct bends achieved by gendarmes and to compare them to safe bends. It can also be used for the initial training and retraining in order to better train riders to learn and estimate a safe trajectory.

As a perspective to this work, the proposed algorithm could be investigated to:

- Identify areas for the design and/or assessment of driving assistance devices dedicated to PTWs.
- Improve the positioning accuracy more by combining models (GPS-RTK, IMU/odo).
- Test and validate our proposed algorithm in M-ABS and MTC (motorcycle traction control) systems.

## Figures and Tables

**Table 1:** Comparison of the signal-to-noise ratio (SNR) of the three filters.

SNR (dB)	Wavelet Filter	Median Filter	Butterworth Filter
Ax	4.77	3.83	2.26
Ay	8.65	7.13	3.91
Az	27.41	19.51	11.29
Rx	1.45	0.42	0.13
Ry	3.80	2.90	1.74
Rz	0.24	0.05	0.01
Mx	1.18	1.20	1.02
My	0.27	0.18	0.19
Mz	2.19	2.04	1.83
Handelbar	0.38	0.04	−0.17
Laser	0.38	0.35	0.17

**Table 2:** Resulting precision of trajectory reconstruction methods.

Method		Error RMS
Kinematic Models	Bicycle Model	90.86 m
	Cossalter Model	26.70 m
INS		83 m
GPS		1.42 m
GPS/INS		0.51 m
INS/Odo		49.39 m

**Table 3:** Experimental results of the enhanced INS/Odo and INS/GPS methods for four bends.

	Bend 1		Bend 2		Bend 3		Bend 4	
	Enhanced INS/Odom	INS/GPS	Enhanced INS/Odom	INS/GPS	Enhanced INS/Odom	INS/GPS	Enhanced INS/Odom	INS/GPS
Bias error	0.3077	0.6493	0.3632	0.8315	0.2300	0.7893	0.2361	0.6960
Error variance	0.1869	0.3456	0.3138	0.4462	0.2520	0.4194	0.2605	0.3908
Max error	0.5603	1.1426	0.8941	1.2192	0. 6927	1.1210	0.6927	1.3085
Improvement ratio	53%		56%		71%		66%	
